# The Fujian eye cross sectional study: objectives, design, and general characteristics

**DOI:** 10.1186/s12886-022-02346-6

**Published:** 2022-03-11

**Authors:** Yang Li, Qinrui Hu, Xiaoxin Li, Yonghua Hu, Bin Wang, Xueying Qin, Tao Ren

**Affiliations:** 1 Eye Institute and Affiliated Xiamen Eye Center of Xiamen University, School of Medicine, Xiamen University, Xiahe Road 336, Siming District, Xiamen, 361003 Fujian China; 2 Fujian Provincial Key Laboratory of Corneal & Ocular Surface Diseases, Xiamen, 361002 Fujian China; 3 Department of Epidemiology and Biostatistics, School of Public Health, Peking University Health Science Centre, Xueyuan Road 38, Haididan District, Beijing, 100191 China; 4 Peking University People’s Hospital, Xizhimennei Street 44, Xicheng District, Beijing, 100044 China

**Keywords:** Cross sectional, Epidemiology, Urban and rural, Coastal and inland, Eye diseases, Related factor, Visual acuity, Vision impairment

## Abstract

**Purpose:**

To describe the objective and design of the Fujian Eye Study and to introduce the general characteristics and vision condition of this study.

**Methods:**

The Fujian Eye Study (FJES) is a population-based cross-sectional survey on the public eye health status of residents over 50 years old in the entire Fujian Province of Southern China, which contains both urban and rural areas and coastal and inland regions. 10,044 participants were enrolled using a two-stage cluster sampling design and underwent a questionnaire and a series of standard examinations both physical and ocular. The main subgroups of data collection included age, sex, region, refractive error, education background, income, eating habits, smartphone usage in the dark, complaints of eye discomfort, history of chronic diseases, consumption of tobacco, alcohol, or tea.

**Results:**

8211 (81.8%) participants were finally included and were divided into urban populations (4678 subjects) and rural populations (3533 subjects) and coastal residents (6434 subjects) and inland residents (1777 subjects); 4836 participants were female. The mean age was 64.39 (SD 8.87) years (median 64 years; range 50–98 years). 227 (3.33%) had vision impairment (VI), 195 (2.87%) had low vision and 14 (0.21%) were blind. The mean presenting near visual acuity (PNVA) was 0.28 (0.17), the mean presenting distance visual acuity (PDVA) was 0.61 (0.30), and the mean best corrected visual acuity (BCVA) was 0.82 (0.28).

**Conclusions:**

The FJES collected detailed questionnaire information and overall ocular and physical examinations, which provide the opportunity to identify risk factors and images of VI and eye diseases and to evaluate their associations with chronic diseases and basic personal information.

## Introduction

Population-based studies could usually deliver the evidence for findings and hypotheses formulated on the basis of hospital-based investigations and provide new exploration directions and practical basis for basic experimental research. Previous major population-based studies in the field of Ophthalmology were mostly from United States [1, 2], Australia [3, 4], Western Europe [5, 6], South America [7], Middle East [8], Singapore [9, 10] and Japan [11]. China has a large population and great geographical differences. Although there have been several population-based surveys conducted in China, these have primarily been conducted in inland cities or nearby areas or coastal metropolitan area [12–14]. Eighty percent of Fujian Province is in a mountainous area, where transportation is inconvenient and economic and medical resources are limited. The lack of eye health data in Fujian Province attracted the attention of a National Natural Science Foundation of China (NSFC) applicant. Due to the specific geographical advantages of this location, we can obtain valuable ophthalmic survey data that may be a main focus of future attention.

The Fujian Eye Study (FJES) is a population-based cross-sectional on-site survey of eye health projects in public health of more than 10,000 residents in Fujian Province, southeastern China, which was part of the national “active health and aging science and technology response” key project. Our team crossed over 20,000 km and performed a field survey of more than 50 towns that covered both urban and rural areas and coastal and inland regions. This is the first ophthalmologic epidemiological survey of an entire coastal province of China to date. Previous eye surveys in China were in northern areas [12, 13], urban areas of southern China [14], eastern areas [15] and a national survey in nine inland provinces [16]. Our study was designed to examine the prevalence, relative factors and impact of eye diseases in noninstitutionalized, community-dwelling persons aged 50 years or older in Fujian Province to determine both modifiable and nonmodifiable risk factors that may be associated with ocular diseases and to understand the differences in and barriers to eye care services in these coastal and inland regions. At the same time, lectures about eye health and medical consulting services were provided for residents.

## Methods

### Study design

**Fig. 1:**
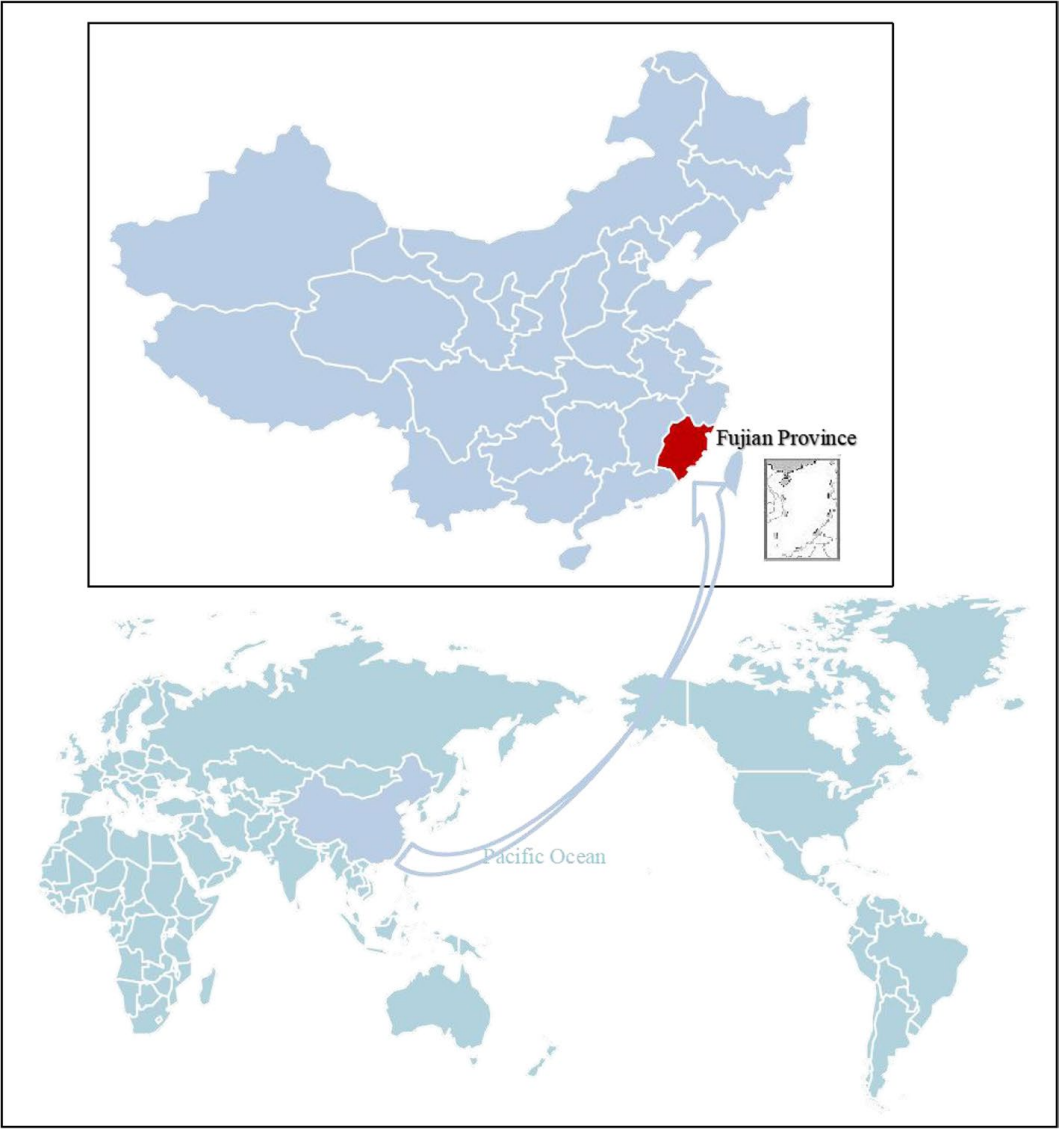
The geographical location of the Fujian Eye Study

**Fig. 2:**
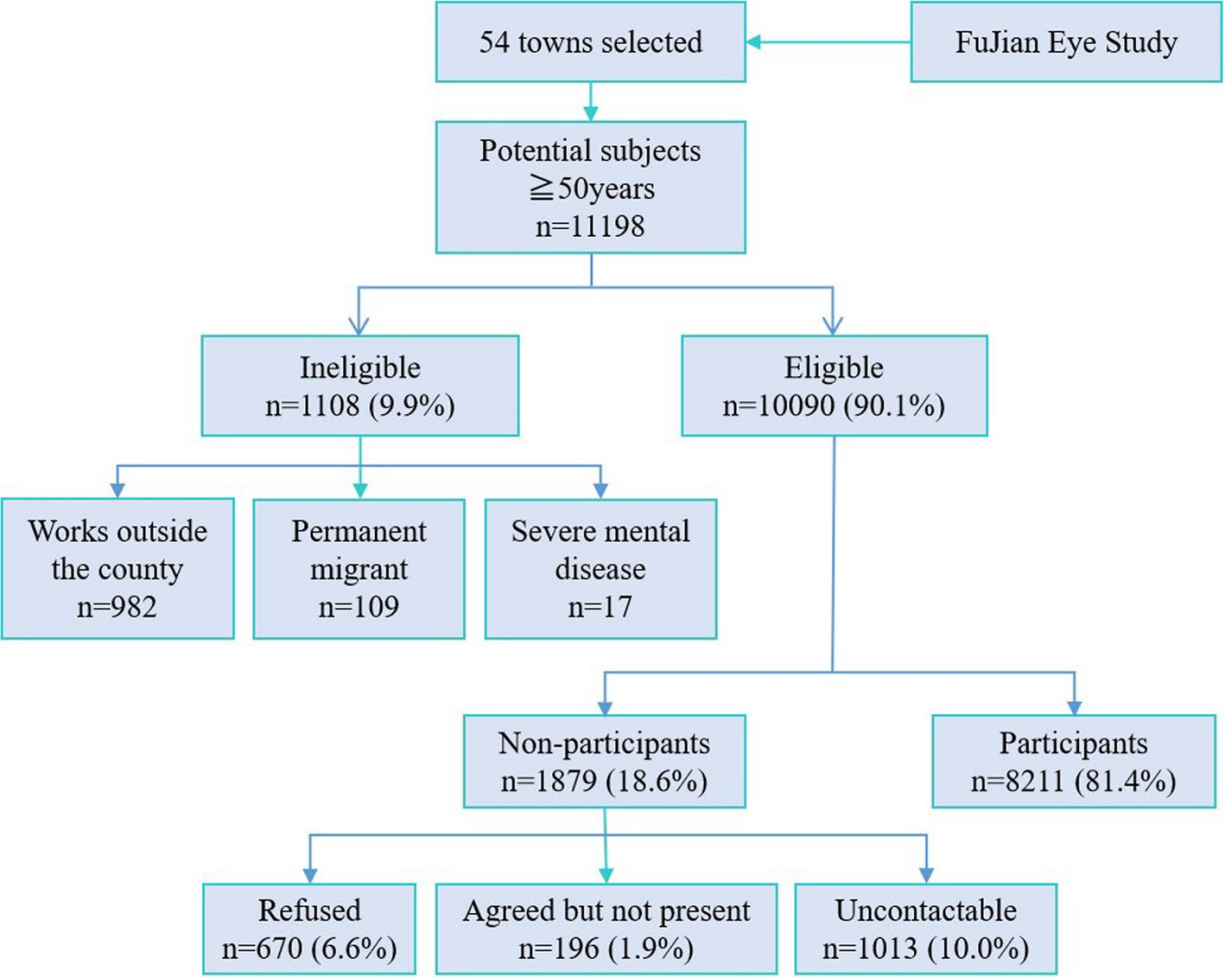
Flowchart of recruitment in the Fujian Eye Study

The FJES was performed from May 2018 to October 2019 as an ophthalmologic epidemiologic on-site survey on a random sample of coastal and inland, urban and rural Southern Chinese adults, reflecting the current eye health status of local Chinese people. The location of the study is illustrated in Fig. 1. We aimed to examine the distribution, trends and risk factors for eye diseases between different areas to explore the influence of genetic and environmental factors and the appropriate intervention measures for eye diseases. This research involved the digitization of image data, standardization of diagnosis, assistance by artificial intelligence and a combined study of genetic and environmental factors. It provides an overview of indigenous physical and eye health and is beneficial for global health management. A clinical study registry was obtained for the 2018–2019 FJES study (register number: ChiCTR2100043349) and the study protocol was approved by the Ethics Committee of Xiamen Eye Center affiliated with Xiamen University (Acceptance number: XMYKZX-KY-2018-001), and written informed consent was obtained from all participants. All residents in a community are officially registered at the local mayor’s office. Using this register as the sampling frame, all residents of the communities who were aged over 50 years were eligible for the study. We used a two-stage cluster sampling design where the clusters were first selected with probability proportional to cluster number, and then subjects are randomly sampled inside selected clusters. According to the data of the National Bureau of Statistics and Fujian Provincial Department Of Finance in 2017 (http://czt.fujian.gov.cn/zfxxgk/fdzdgknr/czzjgl/zjfpwj/201803/t20180329_4563507.htm), the permanent residents of Fujian Province was approximately 38.74 million and there were 2153 communities. Our finally total sample was 10,044 subjects, so we randomly selected 54 communities in an equal proportion manner, including 33 urban communities and 21 rural communities. Then we randomly selected subjects based on the register from each community according to the community size. Figure 2 summarizes the survey design and implementation details for the completed survey administration, including the survey field period, survey mode, total sample size and final sample size.

### Inclusion and exclusion criteria

#### Inclusion criteria

I. Officially registered at the local mayor’s office
II. Age over 50 years
III. Live and work in the county right now
IV. Good mental state, and able to cooperate with the examination

#### Exclusion criteria

I. Not officially registered at the local mayor’s office
II. Age less than 50 years
III. Not live or work in the county right now
IV. Permanent migrant
V. Have severe mental disease which can not cooperate the inspection

### Sample size considerations

The following calculation formula was used to estimate the sample size: *n* = deff×μ_α_^2^ × *p* × (1-*p*)/d^2^. The present study can achieve a precision of 0.05 (d), considering confidence interval of 95% (bilateral), μ_α_^2^ of 1.96, design effect of 2, relative error of 0.15 and d = r × *p*. The sample size was targeted to achieve an adequate precision around estimates of prevalence and to allow for risk factor analyses to be carried out. As the prevalence of the main eye diseases in cross-sectional baseline surveys were estimated to be greater than 2.0% [6, 17–20]. Based on the data above and the response rate of pilot study (about 85%), 10,044 subjects would be recruited in this study. According to the data of the National Bureau of Statistics in 2017 [21], the permanent population of Fujian Province was about 38.74 million with 14.0% aged 50 to 59 years, 10.0% aged 60 to 69 years, 4.3% aged 70 to 79 years, and 1.8% aged 80 years and older, including 24.64 million urban population and 14.1 million rural population (58.1%: 41.9%) or a coastal population of 30.90 million and an inland population of 7.84 million (79.8%: 20.2%). Therefore, a total of 5836 urban and 4208 rural residents or 8015 coastal and 2029 inland residents were needed.

### Survey constructs and measures

**Fig. 3:**
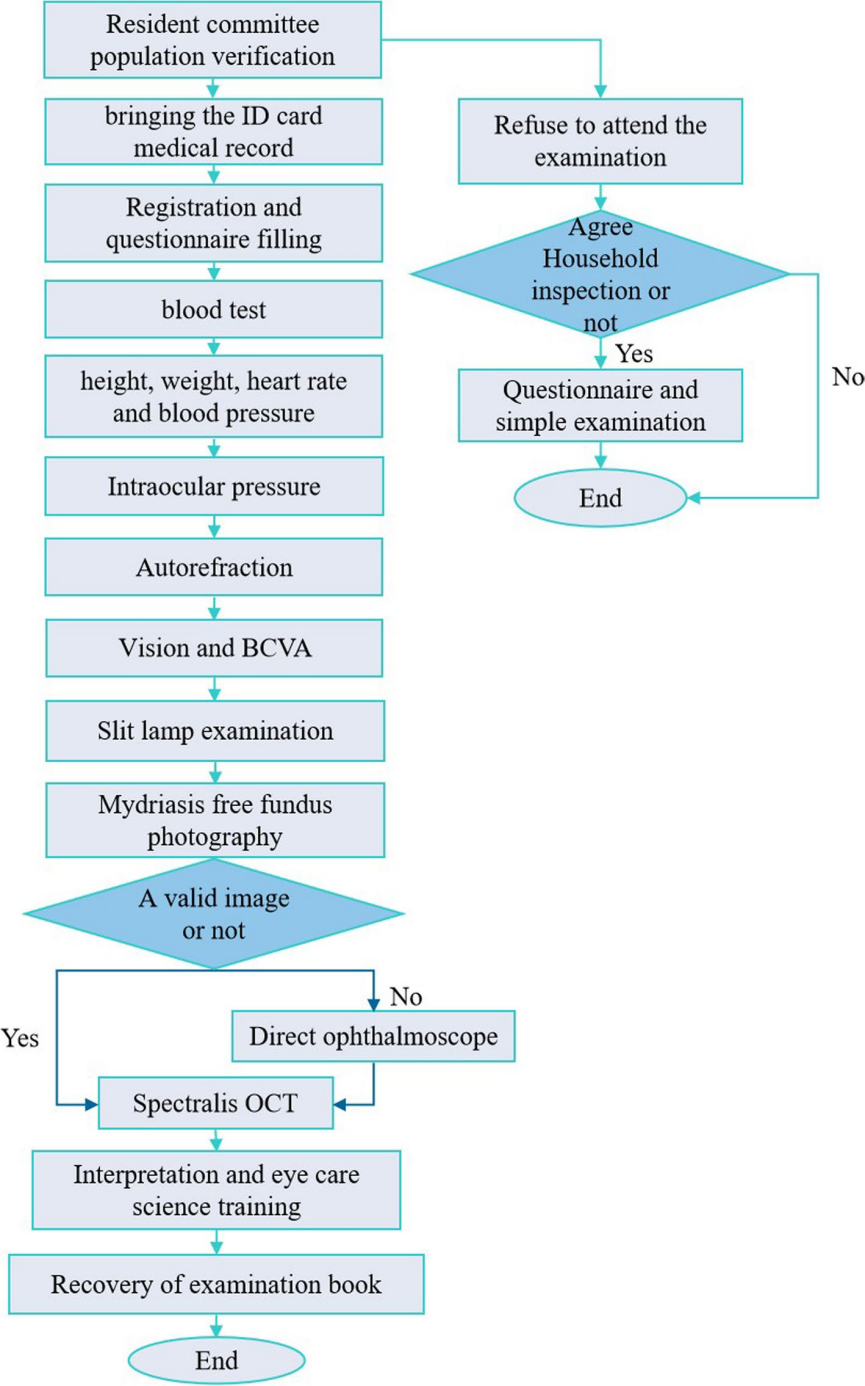
Flowchart of on site inspection in the Fujian Eye Study

The FJES provides exhaustive longitudinal information including sociodemographic and administrative data (sex, age, nationality, etc.), physical data (height, weight, body mass index (BMI), heart rate (HR), systolic blood pressure (SBP), diastolic blood pressure (DBP), blood index) and ocular data (presenting near visual acuity (PNVA); presenting distance visual acuity (PDVA); refractive state; best corrected visual acuity (BCVA); intraocular pressure (IOP); slit lamp examination and fundus examinations (nonmydriatic color fundus photography and multicolour optical coherence tomography (OCT)) data. The process of on-site inspection is shown schematically in Fig. 3.

PDVA and BCVA were measured using E Standard Logarithmic Visual Acuity Chart (GB 11533—1989) at a distance of 5 m. For results less than 0.1, PDVA and BCVA at a distance of 1 m were tested. If that was not possible, finger counting, hand movement, and light perception were tested.

NVA was measured by logarithmic visual acuity chart at a distance of 30 cm, followed the WHO definitions of VI as BCVA in better eyes of < 20/60 or worse (equaled with 0.3 in E Standard Logarithmic Visual Acuity Chart (GB 11533—1989)) and blindness as BCVA in better eyes worse than 20/400 (equaled with 0.05 in E Standard Logarithmic Visual Acuity Chart (GB 11533—1989)), defined PNVI as PNVA worse than 20/50 (equaled with 0.4 in our logarithmic near visual acuity chart) and defined presbyopia as PNVA worse than 20/50 and BCVA better than 20/40 (equaled with 0.5 in E Standard Logarithmic Visual Acuity Chart (GB 11533—1989)).

The Lens Opacities Classification System III (LOCS III) was used to evaluate the type of cataract. It consisted of six slit-lamp images for grading nuclear colour (NC) and nuclear opalescence (NO), five retroillumination images for grading cortical cataracts (C), and five retroillumination images for grading posterior subcapsular (P) cataracts [22].

A fundus photograph of each eye was taken using a scanning laser device (Digital Fundus Camera, VISUCAM 524, Goeschwitzer Strasse 51–52, 07745 Jena, Germany), which has a resolution of 20 μm and is able to capture the fundus even through an undilated pupil. Fundus photography was used to take at least two fundus photographs following the operation rules, one centred on the macular disc and one centred on the optic disc.

Multicolour OCT (Spectralis OCT, Heidelberg Engineering GmbH 69,121, Heidelberg, Germany) was used for high-resolution imaging of the optic disc and central retina in both eyes. Prior to imaging, autokeratometry data were entered to correct for ocular magnification effects. The instrument’s eye-tracking software was used to minimize the effects of eye movements. If a lesion was visible, the lesion site was identified with multi-slice scanning images. The protocol for Multicolour OCT imaging for each eye was as follows [23].

#### Disc-centred scans

Forty-nine-line raster scan of a 15° × 10° area. The average of nine frames for each B-scan was used to improve quality.

Peripapillary retinal nerve fibre layer (RNFL) thickness measurements. A circular B-scan of the peripapillary RNFL was taken along a 3.5 mm-diameter (~ 12°) circle.

Optic nerve head radial and circle (ONH-RC) scan. Forty-eight equidistant (7.5° spaced) radials and three circle B-scans were obtained. Each radial B-scan was averaged from 25 frames and spanned 4.7 mm. The three circular B-scans were 3.5 mm (~ 11.5°–12.5°), 4.1 mm (~ 13.5°–14.5°) and 4.7 mm (~ 15.5°–16.5°) in diameter, and each was averaged from 100 frames. Prior to starting the scans, the ONH-RC programme automatically detected the foveal and Bruch’s membrane opening positions. The examiner checked and, if necessary, manually corrected the positions of these landmarks.

#### Foveal-centred scans

Thirty-one-line raster scan of a (30° × 25°). Each B-scan was averaged from nine frames.

Enhanced depth imaging of the macular. Each B-scan spanned approximately 8.6 mm (~ 30°), and the average of 100 frames for each scan was recorded for analysis.

### Data quality

Before the on-site survey, all technicians and clinicians recruited were trained uniformly and needed to finish an examination, and each survey examination was required to be fixed consistently with the same technician. During our on-site survey, participants were asked to complete all the tests before they can get the final diagnosis report, in order to improve the response rate. In the aspect of data collation, double entry with EpiData v3.1 (EpiData for Windows, version 3.1, the EpiData Association, Denmark, Europe) was used to check the data to ensure the correctness of the data.

### Data analysis

Stata/SE statistical software (Stata for Windows, version 15.1, StataCorp LLC, Lakeway Drive, College Station, TX, USA) was used to analyse the data. Data are provided as the mean ± standard deviation (SD). Only one randomly selected eye per subject was obtained for statistical analysis of visual acuity unless intraindividual intereye differences were evaluated. Analysis of variance (ANOVA) was applied to compare the mean among groups of normally distributed parameters. Chi-square (χ^2^) tests were used to compare proportions. Multiple regression models were used to examine the relation between visual acuity measurements and selected sociodemographic characteristics. Logistic regression was applied for the comparison of binary parameters versus categorical parameters and for the comparison of binary parameters versus continuous normally distributed parameters. Linear regression was applied for the comparison of normally distributed parameters. Logistic regression was used to examine the correlation degree of each group. The statistical correlations was reported as the correlation coefficient *r* and statistical strength of correlations was described using odds ratio (OR). Confidence intervals (CI, 95%) are presented. All described associations were derived from the multivariable statistical analysis, unless indicated otherwise. All *P*-values were two sided and less than 0.05 were considered statistically significant.

## Results

A total of 8211 residents (response rate, 81.8%, 8211 out of 10,044) aged ≥50 years were eventually included, and 4836 (58.9%) were female. 4678 (57.0%) were from urban area, and 3533 (43.0%) were from rural area. 6434 (78.4%) were from coastal region, and 1777 (21.6%) were from inland region. The response rates were 80.2 and 84.0% for the urban population and rural population, respectively, and 80.3 and 87.6% for the coastal population and inland population, respectively. Mean age was 64.39 (SD 8.87) years (median 64 years; range 50–98 years). The proportion of age group population in this study was similar to that in China on the whole. Per Capital Annual Net Income in this rural area is 16,335 Yuan, approximately $2419 US, which is higher than the average annual income (11,969 Yuan, approximately $1773 US) per Capital of those living in rural areas throughout Mainland China, and 39,001 Yuan in urban area, approximately $5776 US, which is also higher than the average annual income (33,834 Yuan, approximately $5011 US) per Capital of those living in urban areas throughout Mainland China [24]. Table 1 summarizes the general information of the survey, including the total sample size, gender, region, age group, height, weight, body mass index (BMI), heart rate (HR), systolic blood pressure (SBP), diastolic blood pressure (DBP), refractive error, education and income in more detail.

**Table 1:** Composition of the study population

	Total study	Urban population	Rural population	***P*** value	95% CI or χ^2^	Coastal population	Inland population	***P***^***§***^ value	95% CI or χ^2^
Number (subjects)	8211	4678	3533			6434	1777		
Sex									
Female	4836	2697	2139			3804	1032		
Male	3375	1981	1394			2630	745		
Age (years)	64.39 (8.87)	64.64 (8.66)	64.05 (9.12)	0.0028	0.2032 to 0.9775	64.49 (8.74)	64.00 (9.30)	0.0381	0.0272 to 0.9585
Median	64	64	64			64	63		
Range	50 to 98	50 to 98	50 to 95			50 to 98	50 to 93		
Age group (%)									
50 to 54	15.2	13.83	17.01	< 0.0001	32.59	14.45	17.9	< 0.0001	33.52
55 to 59	16.86	16.14	17.8			16.34	18.74		
60 to 64	19.45	20.16	18.51			20.13	16.99		
65 to 69	20.34	21.44	18.88			21.01	17.9		
70 to 74	14.54	15.24	13.61			14.78	13.67		
75 to 79	7.39	6.95	7.98			7.3	7.71		
80+	6.22	6.24	6.2			5.98	7.09		
Height (cm)	160.34 (7.97)	160.69 (8.02)	159.88 (7.89)	< 0.0001	−1.1619 to −0.4578	160.32 (8.06)	160.42 (7.66)	0.6512	−0.3289 to 0.5260
Weight (cm)	61.43 (10.09)	61.86 (10.14)	60.84 (9.99)	< 0.0001	−1.4635 to − 0.5721	61.63 (10.14)	60.67 (9.86)	0.0005	−1.4960 to − 0.4147
BMI	23.85 (3.26)	23.91 (3.21)	23.77 (3.33)	0.0471	−0.2905 to − 0.0019	23.94 (3.29)	23.52 (3.14)	< 0.0001	−0.5861 to − 0.2365
HR (beats/min)	79.08 (11.07)	78.78 (10.83)	79.48 (11.37)	0.0048	0.2151 to 1.1966	79.12 (11.01)	78.94 (11.29)	0.5653	−0.7702 to 0.4209
SBP	136.05 (21.24)	134.27 (20.01)	138.43 (22.57)	< 0.0001	3.2291 to 5.0956	136.35 (21.30)	134.91 (20.99)	0.0130	−2.5784 to −0.3033
DBP	75.83 (12.54)	75.46 (12.24)	76.33 (12.91)	0.0022	0.3101 to 1.4167	75.97 (12.69)	75.33 (11.95)	0.0641	−1.3061 to 0.0373
IOP	13.88 (3.46)	13.74 (3.41)	14.06 (3.50)	< 0.0001	0.1754 to 0.4780	13.77 (3.43)	14.29 (3.52)	< 0.0001	0.3396 to 0.7035
Refractive error	0.52 (2.73)	0.51 (2.69)	0.54 (2.78)	0.68	−0.1480 to 0.0966	0.62 (2.67)	0.18 (2.90)	< 0.0001	0.2886 to 0.5835
Median	1	1	1			1	0.75		
Range	−23.75 to + 14.50	−23.25 to + 14.50	− 23.75 to + 13.25		−23.25 to + 14.50	− 23.75 to + 7.25			
Refraction group (D) (%)									
< −10.00	1.18	1.09	1.3	0.53	7.05	1.07	1.58	< 0.0001	44.28
− 10.00 to −6.00	1.24	1.43	0.99			1.1	1.74		
− 6.00 to −3.00	3.47	14.22	3.4			2.97	5.29		
− 3.00 to 0.00	14.27	14.43	14.07			13.94	15.48		
0	3.99	3.93	4.08			3.9	4.33		
0.00 to + 3.00	66.56	67.4	65.44			68.06	61.11		
+ 3.00 to + 5.00	3.9	3.74	4.1			3.99	3.55		
+ 5.00 to + 10.00	0.54	0.51	0.57			0.56	0.45		
> + 10.00	0.05	0.02	0.08			0.06	0		
Missing data	4.8	3.91	5.97			4.34	6.47		
Level of education (%)									
Illiteracy	15.59	10.3	22.59	< 0.0001	262.69	17.94	7.09	< 0.0001	53.59
Primary school	18.52	16.03	21.82			20.33	11.99		
Middle school	37.72	38.24	37.02			40.95	26		
College and above	14.53	17.34	10.81			14.8	13.56		
Missing data	13.64	18.08	7.76			5.98	41.36		
Income (%)									
< =2000	29.44	25.16	35.1	< 0.0001	222.86	32.45	18.51	< 0.0001	68.68
2000–5000	23.72	29.22	16.44			23.31	25.21		
> 5000	7.21	8.49	5.52			7.82	5.01		
Missing data	39.63	37.13	42.94			36.42	51.27		

*BMI* body mass index, *HR* heart rate, *SBP* systolic blood pressure, *DBP* diastolic blood pressure, *IOP* intraocular pressure, *D* diopter, *CI* confidence intervals, χ^2^ the valve of Chi-squaire analysis

*P* Value, statistical significance of the difference between urban population group and rural population group; *P*^*§*^ Value, statistical significance of the difference between coastal population group and inland population group

In the FJES, 4836 were female, and 3375 were male. Among them, 4776 females and 3287 males had PDVA test results, 4257 females and 2566 males had BCVA results, and 4836 females and 3192 males had PNVA results. The mean PNVA was 0.28 (0.17), the mean presenting VA was 0.61 (0.30) and 0.23 (0.27) logMAR units, and the mean BCVA was 0.82 (0.28) and 0.08 (0.19) logMAR units. Table 2 shows the response rate of best corrected visual acuity by age among the study population in geographical populations. Table 3 reveals the frequency of uncorrected distance visual acuity and best corrected visual acuity in the better eye in this study. Based on the WHO definitions using BCVA, 227 (3.33%) had DVI, 195 (2.87%) had low vision and 14 (0.21%) were blind. 5509 (68.58%) had PNVI, 4279 (68.33%) were noted presbyopia, and 193 (2.85%) had combined both PNVI and DVI. Table 4 shows the VI number of different subgroups.

**Table 2:** Response rate of best corrected visual acuity by age in the Fujian Eye Study

Area	Age (years)	Registered		Examined		Response rate (%)		Region	Age (years)	Registered		Examined		Response rate (%)	
		Male	Female	Male	Female	Male	Female			Male	Female	Male	Female	Male	Female
Urban	50–54	211	603	164	450	77.70%	74.60%	Coastal	50–54	313	830	242	641	77.30%	77.20%
	55–59	247	737	195	528	78.90%	71.60%		55–59	343	982	262	735	76.40%	74.80%
	60–64	372	845	260	545	69.90%	64.50%		60–64	471	1146	347	842	73.70%	73.50%
	65–69	438	838	309	563	70.50%	67.20%		65–69	568	1100	425	903	74.80%	82.10%
	70–74	350	518	242	356	69.10%	68.70%		70–74	445	688	332	500	74.60%	72.70%
	75–79	187	229	123	148	65.80%	64.60%		75–79	275	313	201	219	73.10%	70.00%
	80+	176	197	101	111	57.40%	56.30%		80+	215	266	140	171	65.10%	64.30%
Total		5948		4095		68.80%		Total		7955		5880		73.90%	
Rural	50–54	206	486	176	436	85.40%	89.70%	Inland	50–54	104	259	98	245	94.20%	94.60%
	55–59	225	506	184	436	81.80%	86.20%		55–59	129	261	117	229	90.70%	87.70%
	60–64	222	535	191	484	86.00%	90.50%		60–64	123	234	104	187	84.60%	79.90%
	65–69	257	518	213	449	82.90%	86.70%		65–69	127	256	97	209	76.40%	81.60%
	70–74	210	337	182	300	86.70%	89.00%		70–74	115	167	92	136	80.00%	81.40%
	75–79	156	175	131	151	84.00%	86.30%		75–79	68	91	53	80	77.90%	87.90%
	80+	118	148	94	115	79.70%	77.70%		80+	79	79	55	55	69.60%	69.60%
Total		4099		3542		86.40%		Total		2092		1757		84.00%

**Table 3:** Number of subjects stratified into groups of uncorrected distance visual acuity (*n* = 8178) and best corrected visual acuity (*n* = 6823) in the better eye in the Fujian Eye Study

E chart	Visual acuity		Best corrected visual acuity	
	Frequency	Cumulative percentage	Frequency	Cumulative percentage
0	2	0.02	2	0.03
LP	4	0.07	4	0.09
HM	12	0.22	9	0.22
FC	9	0.33	4	0.28
0.01	5	0.39	2	0.31
0.02	19	0.62	7	0.41
0.03	1	0.64		
0.04	27	0.97	3	0.45
0.05	5	1.03	1	0.47
0.06	30	1.39	5	0.54
0.07	1	1.41		
0.08	25	1.71	9	0.67
0.09	2	1.74		
0.1	78	2.69	22	1
0.12	93	3.83	25	1.36
0.15	146	5.61	27	1.76
0.2	140	7.32	43	2.39
0.25	291	10.88	64	3.33
0.3	353	15.2	110	4.94
0.4	572	22.19	194	7.78
0.5	1004	34.47	379	13.34
0.6	1223	49.43	606	22.22
0.8	2125	75.41	256	25.97
1	1669	95.82	4709	94.99
1.2	304	99.54	315	99.6
1.5	36	99.98	26	99.99
2	2	100	1	100

*LP* light perception, *HM* hand movements, *FC* finger counting

**Table 4:** The number and correlation of present near vision impairment, presbyopia, distance vision impairment and combined vision impairment with different subgroups

Number of Impairment		PNVI	χ^2^	***P*** value	presbyopia	χ^2^	***P*** value	DVI	χ^2^	***P*** value	Combined VI	χ^2^	***P*** value
Areas	Urban	3057	10.29	0.001	2292	0.23	0.633	108	3.02	0.082	84	7.42	0.006
	Rural	2452			1987			119			109		
	Coastal	4300	1.34	0.247	3321	1.13	0.287	146	19.95	< 0.001	130	9.54	0.002
	Inland	1209			958			81			63		
	Total	5509			4279			227			193		
Age group (years)	50 to 54	592	334.56	< 0.001	505	274.53	< 0.001	21	205.8	< 0.001	13	219.04	< 0.001
	55 to 59	868			740			11			9		
	60 to 64	1125			910			29			23		
	65 to 69	1244			979			41			34		
	70 to 74	873			662			36			32		
	75 to 79	438			288			32			29		
	80+	369			195			57			53		
	Total	5509			4279			227			193		
Refraction group (D)	< −10.00	60	961.63	< 0.001	26	880.36	< 0.001	23	346.46	< 0.001	15	217.96	< 0.001
	-10.00 to −6.00	52			40			5			3		
	-6.00 to −3.00	103			70			9			6		
	-3.00 to 0.00	428			303			36			29		
	0	143			122			5			5		
	0.00 to + 3.00	4106			3333			43			39		
	+ 3.00 to + 5.00	273			211			6			6		
	+ 5.00 to + 10.00	36			25			3			3		
	> + 10.00	3			0			2			2		
	Total	5204			4130			132			108		
Education	Illiteracy	1049	495.95	< 0.001	794	367.64	< 0.001	80	74.79	< 0.001	75	83.55	< 0.001
	Primary school	1186			879			39			34		
	Middle school	1974			1553			57			49		
	College and above	526			417			10			5		
	Total	4735			3643			186			163		
Income	<=2000	1830	242.02	< 0.001	1293	152.98	< 0.001	86	18.15	< 0.001	79	21.88	< 0.001
	2000–5000	1144			801			32			27		
	> 5000	270			233			7			4		
	Total	3244			2327			125			110		

*D* diopter, *PNVI* present near vision impairment, *presbyopia* uncorrected near visual acuity worse than N6 or N8 at 40 cm and best corrected visual acuity ≥20/40, *DVI* distance vision impairment, *Combined VI* combined vision impairment

## Discussion

The FJES enrolled 8211 participants from May 2018 to October 2019. The participant baseline characteristics and ocular characteristics were reported. Compared with several cross sectional study before [1–16], our study presented a relatively complete picture of the status quo of vision and ocular diseases and the interrelationship between human, geographical and other physical elements, especially chronic diseases and personal information.

As the study reported, the percentage of blindness and VI in our study was 0.21 and 3.33%, respectively, which was lower than the global data from a Lancet review. The review showed the percentage of blindness and VI was approximately 0.49 and 3.69%, respectively, until 2015 [25]. Besides, the difference was not statistically significant between urban and rural areas in the DVI rate, which was not consistent with some studies, such as the Ireland study [26] and Brazilian Amazon Region Eye Survey [7]. It is necessary to explore the reasons and correlations of these results. To explore the causes in more detail, we included many different groups to collect evidence, such as age, sex, refraction, BMI, SBP, educational background, income, residency, urbanization, history of chronic diseases, tobacco consumption, alcohol consumption, and tea consumption.

In this study, VI was significantly correlated with biological and sociodemographic factors, including age, urban and rural regions, coastal and inland areas, educational background, income and refractive error. Sex was not statistically significantly associated with VI after taking into account the interdependency of the parameters.

Encouragingly, many innovative results were found, such as the difference in VI between coastal and inland regions, a higher DVI rate in the inland population, a higher combined VI rate in both rural and inland populations, and NVA improvement in the tea-drinking population. Dramatic changes have taken place in residents’ lifestyle with the coronavirus disease-19 (COVID-19) outbreak. In particular, near vision at all ages has been affected significantly due to the penetration of electronic products when people reduce outdoor social activities. As a result, future epidemiological research may involve changes to models or performance. Our study only examined eye health status before the epidemic and provides a pre-epidemic sample for future research. The project is the first domestic epidemiological study for eye diseases covering coastal and inland areas in both urban and rural regions and provides evidence for the establishment of policy making and control strategy for eye diseases. The FJES can facilitate collaborations in clinical practice for ophthalmologists and cardiologists or endocrinologists.

There are also some interesting findings. For example, compared with the inland population, the coastal population had less myopia and better vision function overall. Potential differences in economic level, education level, transportation, environmental and lifestyle factors, such as the coastal diet preference for seafood and higher UV levels [27, 28] in coastal areas, may be important factors in the development of vision changes. Another interesting finding was that drinking tea may improve near vision but not distance vision. This discovery may indicate a new direction for basic research. What ingredients in tea may affect vision? The correlations of these factors will be elaborated in the following articles in detail.

The FJES has several strengths and some differential features with regard to other information resources. First, a key strength of the FJES database is the detailed questionnaire information and overall ocular and physical examinations collected in more than 50 towns of all nine cities in an entire province of southern China. Second, many cross-sectional studies have provided a description of VA, but most studies have reported NVA and DVA separately. This study integrated both NVA and DVA and explored the influence of geographic factors, including urban and rural and coastal and inland areas, which have not previously been covered. Third, most comparisons in previous studies were based on various baselines among different studies, whereas the data analysis and comparison in this study used a single baseline, making the correlation more convincing. Fourth, we used advanced measurement instruments, such as multicolour OCT and nonmydriatic colour fundus photography. Furthermore, we designed various subgroups to provide more original information, which can update and complement worldwide epidemiological surveys in ophthalmology.

There are some limitations of this study. First, there may be information biases due to absent registration (data completeness). Second, the examination was not perfect. Because of time limitations, we only performed DVA correction (namely, BCVA), and we did not perform NVA correction. Unfortunately, we also did not perform ocular biometry, which can obtain structural parameters such as the refractive power of the cornea, the depth of the anterior chamber, the axial length of the eyeball and the thickness of the lens. Third, data quality may be a strength in some databases but also a weakness for certain data, such as the incompleteness of NVA and DVA data, which could slightly affect the integrity and accuracy of the final diagnosis results.

## Conclusions

The FJES is a population-based cross-sectional on-site survey on the public eye health status of Chinese residents in Fujian province. The rich data collected from the study provide the opportunity to identify risk factors and associations of VI and eye diseases with chronic diseases and basic personal information. This project will be meaningful for guidance in eye health-related policy-making.

## Data Availability

All data generated or analyzed during this study are included in supplementary information files of this published article.

## Abbreviations

WHO: World Health Organization
VI: Vision impairment
NSFC: National Natural Science Foundation of China
FJES: Fujian Eye Study
BMI: Body mass index
HR: Heart rate
SBP: Systolic blood pressure
DBP: Diastolic blood pressure
PNVA: Presenting near visual acuity
PDVA: Presenting distance visual acuity
BCVA: Best corrected visual acuity
IOP: Intraocular pressure
OCT: Optical coherence tomography
DVI: Distance vision impairment
PNVI: Present near vision impairment
LOCS III: Lens Opacities Classification System III
NC: Nuclear color
NO: Nuclear opalescence
C: Cortical cataract
P: Posterior subcapsular
RNFL: Retinal nerve fibre layer
ONH-RC: Optic nerve head radial and circle
SD: Standard deviation
ANOVA: Analysis of variance
χ^2^: Chi-square
OR: Odds ratio
CI: Confidence internal
UV: Ultraviolet
COVID: Coronavirus disease
VA: Visual acuity
NVA: Near visual acuity
DVA: Distance visual acuity
